# Communication tools for end-of-life decision-making in the intensive care unit: a systematic review and meta-analysis

**DOI:** 10.1186/s13054-016-1264-y

**Published:** 2016-04-09

**Authors:** Simon J. W. Oczkowski, Han-Oh Chung, Louise Hanvey, Lawrence Mbuagbaw, John J. You

**Affiliations:** Department of Medicine, McMaster University, Hamilton, ON Canada; Canadian Hospice Palliative Care Association, Ottawa, Canada; Department of Clinical Epidemiology & Biostatistics, McMaster University, Hamilton, ON Canada; Biostatistics Unit, Father Sean O’Sullivan Research Centre, St Joseph’s Healthcare Hamilton, Hamilton, ON Canada

**Keywords:** Communication, End-of-life, Ethics, Palliative care, Critical care

## Abstract

**Background:**

For many patients admitted to the intensive care unit (ICU), preferences for end-of-life care are unknown, and clinicians and substitute decision-makers are required to make decisions about the goals of care on their behalf. We conducted a systematic review to determine the effect of structured communication tools for end-of-life decision-making, compared to usual care, upon the number of documented goals of care discussions, documented code status, and decisions to withdraw life-sustaining treatments, in adult patients admitted to the ICU.

**Methods:**

We searched multiple databases including MEDLINE, Embase, CINAHL, ERIC, and Cochrane from database inception until July 2014. Two reviewers independently screened articles, assessed eligibility, verified data extraction, and assessed risk of bias using the tool described by the Cochrane Collaboration and the Newcastle Ottawa Scale. Pooled estimates of effect (relative risk, standardized mean difference, or mean difference), were calculated where sufficient data existed. GRADE was used to evaluate the overall quality of evidence for each outcome.

**Results:**

We screened 5785 abstracts and reviewed the full text of 424 articles, finding 168 eligible articles, including 19 studies in the ICU setting. The use of communication tools increased documentation of goals-of-care discussions (RR 3.47, 95 % CI 1.55, 7.75, *p* = 0.020, very low-quality evidence), but did not have an effect on code status documentation (RR 1.03, 95 % CI 0.96, 1.10, *p* = 0.540, low-quality evidence) or decisions to withdraw or withhold life-sustaining treatments (RR 0.98, 95 % CI 0.89, 1.08, *p* = 0.70, low-quality evidence). The use of such tools was associated with a decrease in multiple measures of health care resource utilization, including duration of mechanical ventilation (MD −1.9 days, 95 % CI −3.26, −0.54, *p* = 0.006, very low-quality evidence), length of ICU stay (MD −1.11 days, 95 % CI −2.18, −0.03, *p* = 0.04, very low-quality evidence), and health care costs (SMD −0.32, 95 % CI −0.5, −0.15, *p* < 0.001, very low-quality evidence).

**Conclusions:**

Structured communication tools may improve documentation of EOL decision making and may result in lower resource use. The supporting evidence is low to very low in quality. Further high-quality randomized studies of simple communication interventions are needed to determine whether structured, rather than ad hoc, approaches to end-of-life decision-making improve patient-level, family-level, and system-level outcomes.

**Trial registration:**

PROSPERO CRD42014012913

**Electronic supplementary material:**

The online version of this article (doi:10.1186/s13054-016-1264-y) contains supplementary material, which is available to authorized users.

## Background

### Rationale

With its advanced technology, the intensive care unit (ICU) can provide life-saving medical treatment to the sickest of patients; however, that same technology can also prolong the dying process for patients who are unlikely to survive. Furthermore, many people who are approaching the end of life (EoL), either due to advancing age or progressive disease, would opt for fewer invasive and aggressive treatments in favor of a more palliative or comfort-based approach if asked, but such preferences are often undocumented in the medical record [1]. As a result, ICU clinicians and the substitute decision-makers (SDMs) often engage in goals-of-care discussions to interpret the patient’s known values and preferences in the context of their illness, and to decide which ICU treatments would be in keeping with their wishes [2]. Given the medical and moral complexity of such discussions, and the need to conduct them under acute, often stressful conditions, many communication tools (including decision aids, structured meeting plans, and educational interventions) have been developed in order to assist SDMs and clinicians with EoL decision-making.

However, there remains uncertainty as to whether the use of structured communication tools for EoL decision-making is superior to usual care. Therefore, we conducted a systematic review of the medical literature to determine the impact of communication tools for EoL decision-making in the ICU on the following outcomes: the number and quality of EoL discussions between SDMs and healthcare providers (HCPs); the documentation of code status; and decisions to withdraw or withhold life-sustaining treatments.

## Methods

### Protocol and registration

The protocol for this review is available in the PROSPERO registry (http://www.crd.york.ac.uk/PROSPERO/display_record.asp?ID=CRD42014012913).

### Eligibility criteria

We included randomized controlled trials (RCTs) or prospective observational studies with a control group (including pre-post studies in which participants acted as their own control) published as articles in peer-reviewed journals, restricted to the English language. To be eligible for this review, studies must have included patients over the age of 18 years, and evaluated a communication tool to assist in EoL decision-making in comparison to a control group.

For our study, we defined a communication tool as any intervention designed to directly assist individual patients and SDMs in decision-making, or their clinicians to better facilitate the EoL decision-making process. This included traditional decision aids in any format (paper, video, computer, etc.), and other structured approaches to assisting decision-making, including organized meeting plans, consultation with services for the purpose of assisting with decision-making (e.g., ethics or palliative care), and educational interventions on EoL care options. Interventions designed solely for information-sharing (e.g., breaking bad news, providing emotional support) were excluded, because although such interventions may affect decisions at the EoL, it is not their explicit purpose to do so (Table 1). Communication tools for ICU settings are distinct from those in the ambulatory or non-ICU inpatient setting, as they are directed towards patients and SDMs, and not towards patients, who are generally too ill to participate in EoL decision-making; furthermore, communication tools in the ICU setting are usually directed towards decisions about current acute care, rather than advance care planning to prepare for future acute illness. For this reason, we report findings from eligible studies that were conducted in the ICU. Studies conducted in the ambulatory setting and in the inpatient non-ICU setting, and studies of educational interventions for improving clinicians’ competencies in EoL communication and decision-making will be analyzed and reported separately.

**Table 1:** Study eligibility criteria

Eligibility criterion	Rationale
Randomized, controlled trial or prospective observational study, published in peer-reviewed journal	Randomized controlled trials and prospective observational study experimental designs are least likely to lead to biased results
Evaluates a structured communication tool (decision aid, structured meeting, educational strategy) compared to control group	• Interest in comparing wide variety of interventions, in multiple formats (verbal, paper, video, computer, etc.) • A control group is required to assess whether the intervention is better than usual care as routinely practiced (recognizing that usual care may vary based on setting)
Communication tool must address end-of-life decision-making	Review interest is in interventions that assist patients with decision-making, as opposed to those that address breaking bad news, patient comfort alone
Adult patients (age >18 years)	End-of-life decision-making process in frail adults is likely to be qualitatively different from that in children
English language	Communication tools published in other languages, with no English translation available, may not be generalizable to english-language settings

For this paper, which focuses on the ICU setting, our primary outcomes were: (1) proportion of patients with documented goals of care discussions; (2) proportion of patients with documented code status; and (3) proportion of patients with new decisions to withdraw or withhold life-sustaining treatments, as we considered these to be the most immediate patient-level outcomes affected by goals-of-care discussions. Our secondary outcomes were (1) patient or family satisfaction with EoL care; 2) patient or family knowledge about EoL care, including knowledge about palliative care and intensive care; (3) quality of communication between the patient/SDM and HCPs; (4) health care resource utilization (including duration of mechanical ventilation, ICU length of stay, hospital length of stay, and financial cost of care); and (5) the acceptability of the intervention. Secondary outcomes 1–3 were selected to help understand the mechanisms by which communication tools affect EoL decisions, while secondary outcome 4 was chosen to understand the resource implications of using these tools, and, for study participants who received the intervention, secondary outcome 5 was chosen to help understand whether the use of such tools, irrespective of other benefits, would be acceptable to patients/SDMs.

### Information sources and search strategy

We searched the following databases from database inception until the present: MEDLINE (1946 through July 2014); Embase (1980 through July 2014); CINAHL (1982 through July 2014); Cochrane Database of Clinical Controlled Trials (2005 through July 2014); and ERIC (1966 through July 2014). Our search terms included: “communication,” “decision-making,” “end-of-life,” “cardiopulmonary resuscitation” (complete electronic search strategies for each database can be found in Additional file 1: Appendix 1). We also hand-searched the reference lists of eligible articles to identify further articles for screening.

### Study selection

Retrieved titles and abstracts were screened independently and in duplicate by two reviewers (HC, SO) for potential eligibility using standardized, piloted screening forms. The full text of all articles that passed initial screening by either reviewer were then assessed independently and in duplicate using standardized, piloted eligibility forms. Disagreement about study eligibility was resolved by consulting with a third reviewer (JY). Reviewers were not blinded as to article authors, journal, or results, when screening for eligibility. Kappa statistics were calculated to assess the inter-rater reliability of the screening and eligibility phases [3]. Studies were then divided based on study type into outpatient, inpatient, or intensive care unit settings; or educational interventions for clinicians.

### Data collection process and data items

Study data were collected using standardized, piloted online forms by the two reviewers (HC and SO). Study authors were contacted to obtain missing data for our primary or secondary outcomes. We collected data on publication information, study dates and population characteristics, study interventions, our primary and secondary outcome measurements, and the study methods required to assess the risk of bias in individual studies.

### Risk of bias in individual studies

For RCTs, we assessed the risk of bias in individual studies using the Cochrane risk-of-bias tool with regard to random sequence generation, allocation concealment, blinding of participants and personnel, incomplete outcome data, selective reporting, and where appropriate, cluster design [4]. Each domain was assessed independently by both reviewers and reported as having high, low, or uncertain risk of bias. Studies were considered to have low risk of bias if assessed as having low risk of bias in all domains; to have uncertain risk of bias if assessed as having uncertain risk of bias in at least one domain and no domains at high risk of bias; and to have high risk of bias if there was high risk of bias in any domain. For studies with uncertain risk of bias, we attempted to contact study authors to clarify the relevant issue(s), and revised the overall study risk-of-bias accordingly. Disagreement between reviewers about risk of bias was resolved by consulting with a third reviewer (JY). For observational cohort and case–control studies, we used the Newcastle-Ottawa scale to assess risk of bias, using seven stars for our cutoff for good vs. poor studies [5]. For uncontrolled before–after studies, the National Institutes of Health rating system was applied [6].

### Synthesis of results

We used Revman 5.3 software to conduct our analyses [7]. For each outcome, similar studies were pooled, with priority given to randomized trials i.e., data were sought from RCTs first and non-randomized studies were only used in the absence of data from RCTs. However, given the small number of RCTs found, we also reported the results of observational studies in separate analyses for comparison. Pooled outcomes (standardized mean difference (SMD) or mean difference (MD) for continuous variables, relative risk (RR) for dichotomous variables) and 95 % confidence intervals (95 % CI) were calculated using a random-effects model. For cluster-randomized RCTs, intraclass correlation coefficients were used to adjust the effective sample size to adjust for clustering effects within groups [8]. Where standardized deviations were missing, or represented by interquartile ranges, estimates were generated using the calculations described in the Cochrane handbook [9].

### Investigation of heterogeneity and subgroup analyses

Clinical heterogeneity was assessed by reviewers investigating study populations, interventions, and comparisons. If the studies were considered to be of sufficient similarity for data pooling, heterogeneity was quantified for each outcome of interest and using the *I*^2^ statistic, with values greater than 50 % indicating substantial heterogeneity [9].

We conducted one exploratory subgroup analysis, comparing the results of the communication tools between patients who survived to discharge from ICU (ICU survivors) and those who died in the ICU (ICU non-survivors). Several of the studies only reported outcomes for ICU non-survivors. We chose to report these results separately, as the subgroup of ICU non-survivors may represent a population more likely to have withdrawal of life support (due either to pre-existing advance directives, severity of illness, or medical comorbidities), and would thus be inappropriate to pool with studies that included both ICU survivors and non-survivors. This subgroup is also of interest as it may correlate with a group of patients for whom ICU resources constitute ‘non-beneficial care’ [10].

### Publication bias

Publication bias was assessed using visual inspection of funnel plots generated in Revman 5.3, where sufficient numbers of studies existed to permit interpretation [11].

### Rating of quality of evidence

We used the Grading of Recommendations Assessment, Development, and Evaluation (GRADE) approach to assess the quality of evidence for each outcome [12]. Outcomes for which the majority of evidence was derived from RCTs was considered to initially be of high quality, while those from which the majority of evidence was from observational studies started at low quality, with both types rated up or down after considering the risk of bias across studies (e.g., publication bias), potential biases and their direction within each study, and the imprecision, inconsistency, and indirectness of the evidence. GRADE summary of findings tables were generated using the online GradePRO software [13].

## Results

### Study selection

**Fig. 1:**
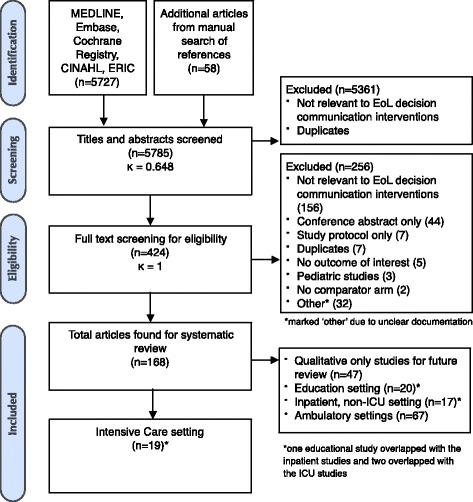
Flowsheet of study screening, eligibility, and inclusion

Initial database searches retrieved 5727 articles, with 58 articles found using reference screening. After exclusion of duplicate references and conference abstracts, title and abstract screening resulted in 424 articles eligible for full text review (κ = 0.648; 95 % CI 0.601, 0.695). A total of 168 articles were eligible for our systematic review after full-text review and additional manual reference screening, of which 19 studies were conducted in the ICU setting (Fig. 1).

### Study characteristics

#### Study settings and populations

Study publication dates ranged from 1995 to 2014. Most were conducted in the USA or Canada (n = 16, 84 %), with three conducted in France (16 %). Three studies were conducted in medical ICUs, two in surgical ICUs, and fourteen in mixed medical-surgical or non-specified ICU type. Seven studies specifically focused on patients with longer ICU or hospital stays [14–20]. Two studies were conducted in patients considered likely to die [21, 22]. One study specifically evaluated interventions in patients for whom there were conflicts over goals of care [23] (Table 2).

**Table 2:** Study characteristics

Study ID	Sample size	Population description	Country	Design	Target of intervention	Intervention	Comparator	Measured outcome
Andereck 2014 [14]	Intervention 174, control 210	Patients admitted to the medical or surgical ICU of a tertiary care hospital for at least 5 consecutive days	USA	RCT	Family/SDM	The intervention group received a proactive ethics consultation. The ethics consultation assessed patient capacity and preferences, and assisted SDMs in medical decision-making, including DNR. The ethicist continued to follow the patient until discharge	Usual care	• Health care resource utilization • Satisfaction with end-of-life care
Lautrette 2007 [21]	Intervention 63, control 63	Adult patients admitted to medical or surgical ICUs judged to be likely to die within a few days, with an identified SDM	France	RCT	Family/SDM	An intensive end-of-life communication intervention aimed at eliciting the patient’s values, acknowledging the family member’s voice and emotions, and to allow questions. Following the meeting, families were given a brochure on bereavement	Usual care	• Quality of communication • Preference on life-sustaining treatment options • Advance directive discussions • Health care resource utilization
Schneiderman 2000 [23]	Intervention 35, control 35	ICU patients in whom value-based treatment conflicts arose (e.g., disagreements over CPR status, withdrawal of life support, etc.)	USA	RCT	Family/SDM	Offering of an ethics consultation from the hospital ethics service	Usual care	• Health care resource utilization • Preference on life-sustaining treatment options • Acceptability of intervention
Schneiderman 2003 [24]	Intervention 278, control 273	Critically ill adult patients admitted to medical or surgical ICUs	USA	RCT	Family/SDM	The intervention group received a proactive ethics consultation, which addressed current ethical issues, reviewed patient wishes and values, and provided recommendations for next steps regarding communication and decision-making	Usual	• Health care resource utilization • Acceptability of intervention
Ahrens 2003 [32]	Intervention 43, control 108	Patients admitted to an academic tertiary care medical ICU	USA	Cohort	Family/SDM	Families/SDMs were provided with an intensive communication strategy, including daily medical updates by the attending physician, provision of treatment options, including non-curative/palliative options, and support by a clinical nurse specialist	Usual care	• Health care resource utilization
Campbell 2003 [29]	Intervention 20, control 18	Patients admitted to the medical ICU with either global cerebral ischemia or multisystem organ failure, with a retrospective control cohort and prospective interventional cohort	USA	Cohort	Family/SDM	Early involvement of palliative care service in communicating prognosis to the family, identifying advance directives and preference, and assisting with discussion and implementation of treatment options and palliative care	Usual care	• Preference on life-sustaining treatment options • Health care resource utilization
Cox 2012 [15]	Intervention 10, control 17	SDMs for adult medical and surgical ICU patients on mechanical ventilation for equal to or greater than 10 days, expected to survive for greater than 72 hours without pre-existing tracheostomy	USA	Cohort	Family/SDM	The prolonged mechanical ventilation decision aid reviewed medical information, elicited the SM understanding of the patient’s preferences, clarified the role of the SDM, and provided guidance in decision-making	Usual care	• Quality of communication • Comfort and confidence (decision conflict) • Health care knowledge and literacy • Health care resource utilization • Preference on life-sustaining treatment options
Daly 2010 [16]	Intervention 354, control 135	Incapable patients with 72 hours of mechanical ventilation, with an identified SDM, admitted to surgical, medical, or neuroscience ICUs at two university-affiliated medical centers	USA, Canada	Cohort	Family/SDM	An intensive communication system, including a family meeting with a medical update, identification of goals of care, a treatment plan, and milestones for determining if the treatment was effective, conducted within 5 days of ICU admission and weekly thereafter.	Usual care	• Preference on life-sustaining treatment options • Health care resource utilization • Quality of communication
Dowdy 1998 [17]	Intervention 31, control 31	Sequential patients treated with mechanical ventilation for more than 96 hours, between June 1992 and October 1994	USA	Cohort	Family/SDM	Proactive ethics consultation, and daily as required, addressing advance directives, patient capacity, SDM knowledge of patient advance directive, anticipated conflicts, and limits of treatment	Usual care	• Preference on life-sustaining treatment options • Health care resource utilization • Quality of communication
Hatler 2012 [18]	Intervention 98, control 105	Patients admitted to a territory neurosurgical ICU who received mechanical ventilation for >96 hours, remained in ICU for 7 days or longer, and were not awaiting transfer out of ICU during that time	USA	Cohort	Family/SDM and HCPs	A surrogacy information and decision-making tool was filled out by the admitting nurse, documenting patient’s decision-making capacity, the identity of the SDM/POA, and prior advance directive. The nurse gave the patient or SDM an information sheet about surrogate decision-making and advance directives.	Usual care	• Health care resource utilization
Holloran 1995 [28]	Intervention 6, control 24	Patients admitted to a large, tertiary care ICU for any reason.	USA	Cohort	HCPs	“Decisions near the End of Life” program, a small-group workshop using cases to facilitate discussion of issues such as withholding or withdrawing treatment, eliciting patient and family wishes, patient competency, and conflict with families	Pre-intervention hospital cohort	• Health care resource utilization • Preference on life-sustaining treatment options
Knaus 1990 [25]	Intervention 705, control 760	All adult patients admitted to ICU, excluding those with uncomplicated myocardial infarction or those admitted with acute burns	France	Cohort	HCPs	HCPs were provided with a calculated estimate of hospital mortality daily on rounds until the patient died, or until 7 days, whichever came first	Usual care	• Preference on life-sustaining treatment options
Lamba 2012 [27]	Intervention 104, control 79	Patients admitted to a surgical ICU between March 2003 and May 2005 for liver transplantation	USA	Cohort	Family/SDM	Each patient had a palliative care assessment delineating prognosis, advance directives, family support, surrogate decision maker, and pain, within 24 hours of admission. The patient’s family received psychosocial and/or bereavement support. An interdisciplinary family meeting was held at 72 hours to address patient outcomes, treatment options, and goals of care, and family support was provided by a multidisciplinary team.	Usual care	• Quality of communication • Preference on life-sustaining treatment options • Advance directive discussions • Health care resource utilization
Lilly 2000 [26]	Intervention 396, control 134	Consecutive admitted to the ICU of a tertiary care teaching hospital	USA	Cohort	Family/SDM	An intensive communication strategy, including a meeting with the attending physician within 72 hours for patients expected to stay >4 days, with predicted mortality >25 %, or change in functional status, unlikely to return to home	Usual care	• Advance directive discussions • Quality of communication • Health care resource utilization
McCannon 2012 [30]	Intervention 27, control 23	Patients admitted to the medical ICU age >50 years, currently incapable, likely to survive >24 hours, with an identified adult SDM.	USA	Cohort	Family/SDM	A 3-minute video decision support-tool was shown which reviewed CPR methods and outcomes, and the care of a sedated, mechanically ventilated patient, within 72 hours of ICU admission	Usual care	• Health care knowledge and literacy • Preference on life-sustaining treatment options • Acceptability of intervention
Norton 2007 [19]	Intervention 126, control 65	Adult patients admitted to a medical ICU with a hospital stay of 10 days, age >80 years, or two or more life-threatening comorbidities	USA	Cohort	Family/SDM	The intervention group had a proactive palliative care consultation, which facilitated decision-making and family member support, and followed the patient until discharge	Usual	• Health care resource utilization
Quenot 2012 [31]	Intervention 823, control 678	All patients who died in the ICU, or in hospital after discharge to another department, during two periods, one before and one after a 2005 French law on end-of-life and patient rights.	France	Cohort	Family/SDM	An intensive communication strategy, including daily meetings with the attending team, modalities for withdrawing and withholding treatment, a special ‘ethics’ section in the chart, and debriefing sessions	Pre-intervention hospital cohort	• Preference on life-sustaining treatment options • Health care resource utilization • Quality of communication
Shelton 2010 [20]	Intervention 114, control 113	Patients admitted to the surgical ICU, anticipated by the attending physician to remain for at least 7 days, or were expected to die within that time, during two periods	USA	Cohort	Family/SDM	During the intervention period, a family support coordinator assessed the family’s information needs, interpreted and explained relevant medical information, assisted the family in decision-making, and identified the need for referrals to spiritual care and to enhance the health care team’s understanding of the family’s needs.	Usual care	• Satisfaction with end-of-life care • Quality of communication with HCPs • Health care resource utilization
Curtis 2011 [22]	Intervention 514, control 565	Medical and surgical ICUs with sufficient ICU deaths to meet study sample size requirements (6 intervention hospitals, 6 control hospitals) Patients included those who died in ICU or within 30 hours of transfer to another hospital location.	USA	Cluster RCT	HCPs	A multifaceted intervention including education about palliative care, identification and training of ICU clinician local champions for palliative care, nurse and physician ICU directors to address barriers to improving end-of-life care, feedback of quality data including family satisfaction, and implementation of system supports such as palliative care order forms.	Usual care	• Satisfaction with end-of-life care • Preference on life-sustaining treatment options • Quality of communication • Health care resource utilization

*RCT* randomized controlled trial, *SDM* substitute decision-maker, *CRP* cardiopulmonary resuscitation, *HCP* health care provider

#### Interventions

Fifteen interventions were directed solely at the family/SDM, three at HCPs, and one intervention had elements directed at both groups. Interventions included ICU team-led intensive communication strategies (n = 6, 32 %); ethics consultations (n = 4, 21 %) or palliative care consultations (n = 3, 16 %) for the purpose of assisting with EoL decision-making; written decision aids (n = 3, 16 %); video decision aids (n = 2, 11 %); and complex multifaceted ICU quality improvement interventions (n = 1, 5 %) (Table 2).

#### Characteristics of excluded studies

Of the studies that underwent full-text review, 256 were excluded because they: did not address EoL decision-making (n = 156, 61 %); were conference abstracts with no corresponding full-text publication available (n = 44, 17 %); were study protocols only (n = 7, 3 %); included pediatric patients (n = 3, 1 %); did not include a comparison group (n = 2, 1 %); or provided only qualitative data (n = 47, 18 %).

### Risk of bias within studies

Five of the included studies were RCTs, of which three were considered to have an overall low risk of bias [14, 21, 24], one to have uncertain risk of bias [23], and one to have high risk of bias [22]. The fourteen observational studies were prospective cohort studies, with four having Newcastle-Ottawa scores considered to be good [16, 17, 25, 26], and the remainder of cohort studies having a poor quality rating (Tables 3 and 4).

**Table 3:**
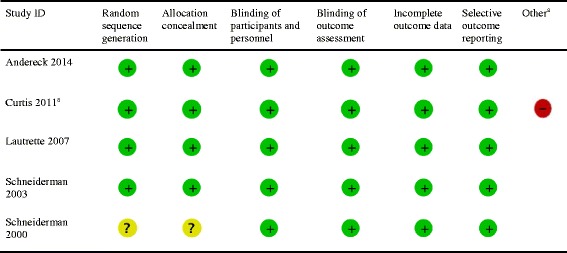
Risk of bias assessment for randomized controlled trials

^a^In a cluster randomized trial, there are other domains to consider for risk of bias, including (1) recruitment bias; (2) baseline imbalance; (3) loss of clusters; (4) incorrect analysis; and (5) comparability. We judged Curtis 2011 [22] to be at high risk due to loss of clusters, incorrect analysis (due to lack of adjustment for clustering effects), and non-comparability between hospitals. Green = low risk of bias, Yellow = unclear risk of bias, Red = high risk of bias

**Table 4:** Risk of bias/quality assessment for observational studies

Study ID	Newcastle-Ottawa scale for cohort studies - selection	Newcastle-Ottawa scale for cohort studies - comparability	Newcastle-Ottawa scale for cohort studies - outcome	Overall Newcastle-Ottawa scale risk of bias
Ahrens 2003 [32]	★★★★	☆☆	★★★	Poor
Campbell 2003 [29]	★★★★	☆☆	★★★	Poor
Cox 2012 [15]	★★★★	☆☆	★★☆	Poor
Daly 2010 [16]	★★★★	★★	★★★	Good
Dowdy 1998 [17]	★★★★	★☆	★★★	Good
Hatler 2012 [18]	★★★★	☆☆	★★★	Poor
Holloran 1995 [28]	★★★☆	☆☆	★★★	Poor
Knaus 1990 [25]	★★★☆	★☆	★★★	Good
Lamba 2012 [27]	★★★★	☆☆	★★★	Poor
Lilly 2000 [26]	★★★★	★☆	★★★	Good
McCannon 2012 [30]	★★★★	☆☆	★★★	Poor
Norton 2007 [19]	★★★☆	☆☆	★★★	Poor
Quenot 2012 [31]	★★★★	☆☆	★★★	Poor
Shelton 2010 [20]	★★★★	☆☆	★★★	Poor

Using the Newcastle-Ottawa Scale, stars are awarded for each quaity item, with the maximum number of stars in the "Selection," "Comparability," and "Outcome" being four, two, and three, respectively. In the table, solid stars indicate stars awarded for quality items, while open stars indicate quality items which were absent

### Synthesis of results

Overall ratings for the quality of evidence for the effectiveness of communication strategies on end-of-life decision-making can be seen in the GRADE summary of findings Tables (Tables 5 and 6).

**Table 5:**
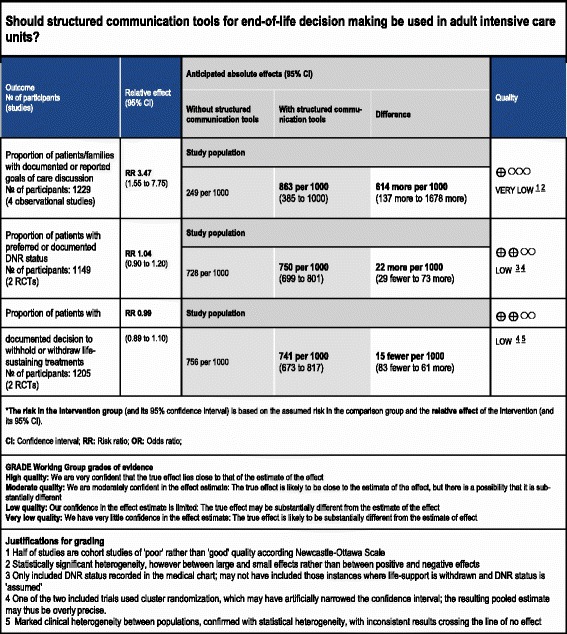
GRADE summary of findings table - primary outcomes

*The risk in the intervention group (and its 95 % confidence interval) is based on the assumed risk in the comparison group and the relative effect of the intervention (and its 95 % CI).

*CI* confidence interval, *RR* risk ratio, *OR* odds ratio, *DNR* do not resuscitate, *RCT* randomized controlled trial

GRADE Working Group grades of evidence

High quality: we are very confident that the true effect lies close to that of the estimate of the effect

Moderate quality: we are moderately confident in the effect estimate. The true effect is likely to be close to the estimate of the effect, but there is a possibility that it is substantially different

Low quality: our confidence in the effect estimate is limited. The true effect may be substantially different from the estimate of the effect

Very low quality: we have very little confidence in the effect estimate. The true effect is likely to be substantially different from the estimate of effect

Justifications for grading

1. Half of studies are cohort studies of poor rather than good quality according to the Newcastle-Ottawa scale

2. Statistically significant heterogeneity; however, between large and small effects rather than between positive and negative effects

3. Only included DNR status recorded in the medical chart; may not have included those instances where life support is withdrawn and DNR status is assumed

4. One of the two included trials used cluster randomization, which have artificially narrowed the confidence interval; the resulting pooled estimate may thus be overly precise

5. Marked clinical heterogeneity between populations, confirmed with statistical heterogeneity, with inconsistent results crossing the line of no effect

**Table 6:**
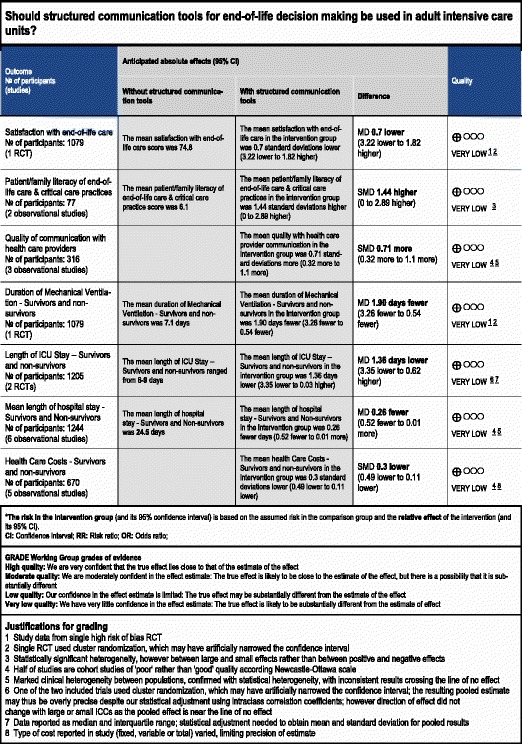
GRADE Summary of findings table - secondary outcomes

*The risk in the intervention group (and its 95 % confidence interval) is based on the assumed risk in the comparison group and the relative effect of the intervention (and its 95 % CI).

*CI* confidence interval, *RR* risk ratio, *OR* odds ratio, *RCT* randomized controlled trial, *MD* mean difference

GRADE Working Group grades of evidence

High quality: we are very confident that the true effect lies close to that of the estimate of the effect

Moderate quality: we are moderately confident in the effect estimate. The true effect is likely to be close to the estimate of the effect, but there is a possibility that it is substantially different

Low quality: our confidence in the effect estimate is limited. The true effect may be substantially different from the estimate of the effect

Very low quality: we have very little confidence in the effect estimate. The true effect is likely to be substantially different from the estimate of effect

Justifications for grading

1. Study data from single RCT with high risk of bias

2. Single RCT used cluster randomization, which may have artificially narrowed the confidence interval

3. Statistically significant heterogeneity; however, between large and small effects rather than between positive and negative effects

4. Half of studies are cohort studies of poor rather than good quality according to the Newcastle-Ottawa scale

5. Marked clinical heterogeneity between populations, confirmed with statistical heterogeneity, with inconsistent results crossing the line of no effect

6. One of the two included trials used cluster randomization, which may have artificially narrowed the confidence interval; the resulting pooled estimate may thus be overly precise despite our statistical adjustment using intraclass correlation coefficients (ICCs); however, the direction of effect did not change with large or small ICCs as the pooled effect is near the line of no effect

7. Data reported as median and interquartile range; statistical adjustment needed to obtain mean and standard deviation for pooled results

8. Type of cost reported in study (fixed, variable or total) varied, limiting precision of estimate

### Primary outcomes

1. Proportion of patients with documented goals of care discussions: one cluster RCT (1079 patients), considered to have a high risk of bias, reported on documented goals-of-care discussions [22], finding a significant reduction in the number of documented discussions in the intervention group (relative risk (RR) 0.82, 95 % CI 0.75, 0.90, *p* < 0.001). By contrast, four observational studies (1229 patients) found an increase in documented goals-of-care discussions (RR 3.47, 95 % CI 1.55, 7.75, *p* = 0.020) [15, 16, 26, 27]. Our overall GRADE assessment for the quality of evidence is very low for both the RCT and observational data, indicating that future studies are highly likely to affect our estimates of effect, for this outcome; we selected the observational studies for our primary analyses, given the limitations of the data from the single RCT (Fig. 2)

**Fig. 2:**
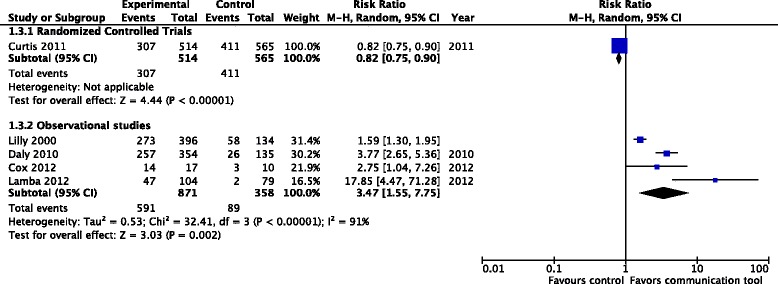
Proportion of patients with documented goals-of-care discussions
2. Proportion of patients with documented code status or ‘do not resuscitate’ (DNR) status: two RCTs (295 patients), one considered to have a high risk of bias [22], the other an uncertain risk of bias [23], reported the proportion of patients with documented code status/DNR status, finding no significant difference with the use of structured communication tools (RR 1.04, 95 % CI 0.90, 1.20, *p* = 0.57, low-quality evidence). Four observational studies (895 patients) also reported on this outcome [16, 17, 27–30], again finding no significant differences between the intervention and control within these studies (RR 1.30, 95 % CI 0.95, 1.78, *p* = 0.11) (Fig. 3)

**Fig. 3:**
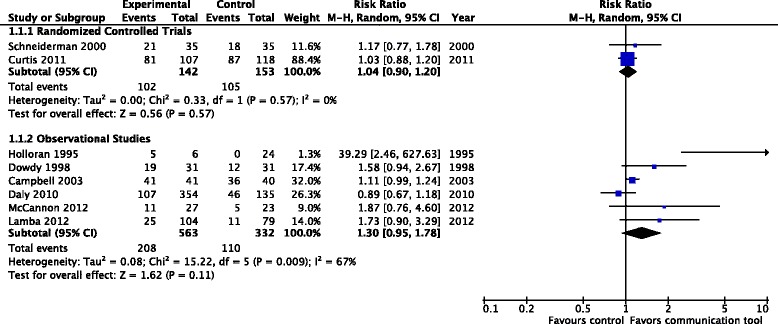
Documented code status/‘do not resuscitate’ status
3. Withholding or withdrawal of life-sustaining treatments: two RCTs (351 patients), one considered to have a low risk of bias [21], and one a high risk of bias [22], reported on decisions to withhold or withdraw life-sustaining treatments, finding no significant difference between the study arms (RR 0.99, 95 % CI 0.89, 1.10, *p* = 0.85, low-quality evidence), although in the study by Lautrette et al. all patients in both arms of the study ended up having life support withdrawn. Six observational studies (3727 patients) also reported on withdrawal of life-sustaining treatments [15–17, 25, 27, 31], finding an increase in treatment withdrawal with the use of communication tools, in contrast to the randomized studies (RR 1.54, 95 % CI 1.2, 1.98, *p* < 0.001); however, there was a large amount of statistical heterogeneity between these studies (*I*^2^ = 99 %) (Fig. 4).

**Fig. 4:**
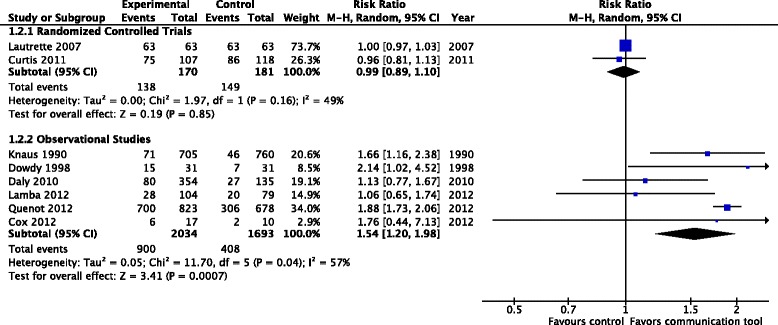
Documented decisions to withdraw or withhold treatments

### Secondary outcomes

1. Patient and family satisfaction with end-of-life care: only one RCT (1079 patients) [22] and one observational study (227 patients) [20] reported on patient and family satisfaction with EoL care. No differences in mean total satisfaction score were seen in the RCT (74.1 (SD 22) vs. 74.8 (SD 20), *p* = 0.59, very low-quality evidence); however, the observational study demonstrated a significant improvement in overall family satisfaction with care (4.5 (SD 0.11) vs. 4.3 (SD 0.3), *p* < 0.001)
2. Family/SDM literacy in end-of-life and critical care practice: two observational studies (77 SDMs) reported measures of SDM literacy of end-of-life care, including understanding of cardiopulmonary resuscitation [30] and mechanical ventilation [15]. SDMs who received the intervention scored significantly higher on tests of medical comprehension (SMD 1.44, 95 % CI 0.0, 2.89, *p* = 0.05, very low-quality evidence) (Fig. 5)

**Fig. 5:**
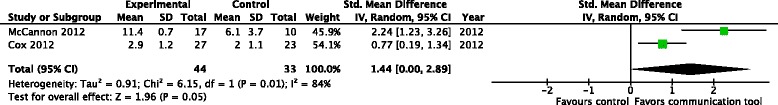
Patient and family literacy in end-of-life care
3. Quality of communication between family/SDMs and the health care provider: one RCT (126 SDMs) reported outcomes related to the quality of communication [21], finding that communication tools resulted in family members reporting an increase in expressing the patient’s wishes (70 % vs. 54 %, *p* = 0.04) and reduction in expressing their own wishes (70 % vs. 84 %, *p* = 0.05), however no summary measures of quality of communication were reported. Three observational studies (316 SDMs) [15, 17, 20] reported measures of quality of communication, finding improved communication scores with the use of structured communication tools (SMD 0.71, 95 % CI 0.32, 1.10, *p* < 0.01, very low-quality evidence) (Fig. 6)

**Fig. 6:**
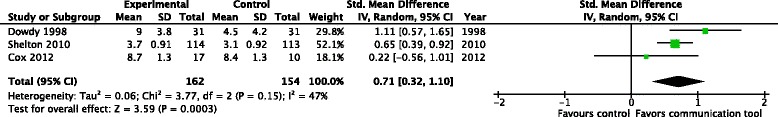
Quality of communication between family/substitute decision-maker and health care providers
4. Health care resource utilization: health care resource utilization was generally reported using one of four measures, which we analyzed separately to ensure clinical interpretability: (a) duration of mechanical ventilation; (b) length of ICU stay; (c) length of hospital stay; and (d) financial costs. A significant number of trials reported health care resource utilization separately for patients who did not survive. We conducted an exploratory subgroup analysis on this subset of patients
  - Duration of mechanical ventilation: one RCT [22] (1079 patients) reported duration of mechanical ventilation, finding a reduced number of days of mechanical ventilation with the use of the communication tool (MD −1.9 days, 95 % CI −3.26, −0.54, *p* = 0.006, very low-quality evidence). Two observational studies [16, 18] (692 patients) also reported on duration of mechanical ventilation but did not note any differences between the treatment groups (MD −0.79 days, 95 % CI −2.21, 0.63, *p* = 0.27) (Fig. 7a)

**Fig. 7:**
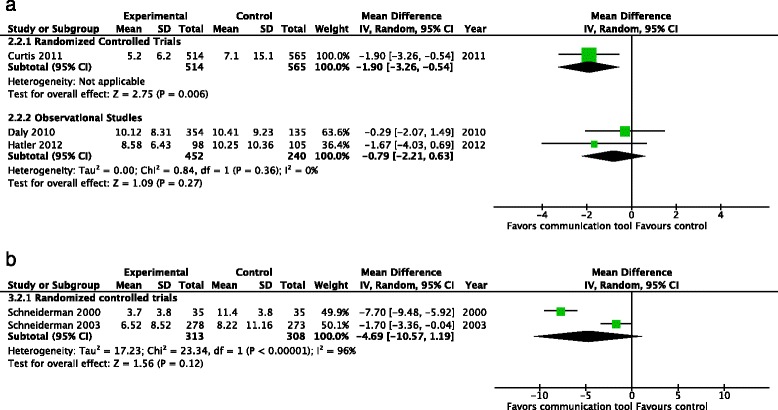
Health care resource utilization - duration of mechanical ventilation (days). **a** Studies including both intensive care unit survivors and non-survivors. **b** Studies including intensive care unit non-survivors only
    Two RCTs (621 patients) reported the effects of the intervention upon duration of mechanical ventilation in ICU non-survivors [23, 24], and did not find a significant reduction in the mean days of ventilation (MD −4.69 days, 95 % CI −10.57, 1.19, *p* = 0.12) (Fig. 7b)
  - Length of ICU stay: two RCTs (351 patients) [21, 22], reported the length of ICU stay, finding a significant reduction (MD −1.36 days, 95 % CI −3.35, 0.62, *p* = 0.18, low-quality evidence), with no evidence of statistical heterogeneity (*I*^2^ = 0 %). Eight observational studies (1824 patients) also reported the length of ICU stay, [15–17, 19, 20, 26, 27, 32], but did not find a reduction with the use of communication tools (MD −1.57 days 95 % CI −3.23, 0.10, *p* = 0.07) (Fig. 8a). Three RCTs (729 patients) [14, 23, 24] reported the length of ICU stay amongst the subgroup of ICU non-survivors, finding no significant reductions with the use of communication tools (MD −3.46, 95 % CI −8.55, 1.64, *p* = 0.18) although significant heterogeneity was noted (*I*^2^ = 96 %). Three observational studies (1625 patients) [17, 29, 31] also reported this outcome, with a significant reduction in the length of ICU stay (MD −5.96 days, 95 % CI −6.51, −5.41, *p* < 0.001) (Fig. 8b)

**Fig. 8:**
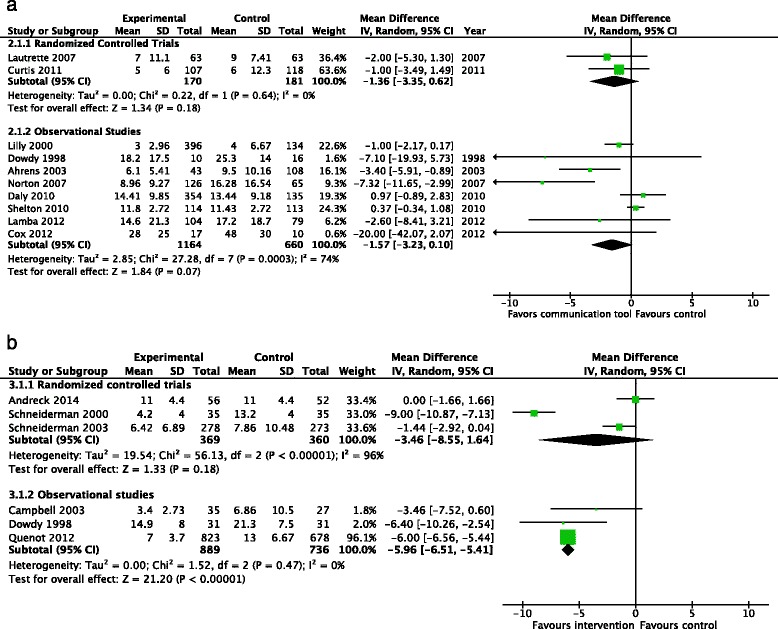
Health care resource utilization - length of intensive care unit stay (days). **a** Studies including both intensive care unit survivors and non-survivors. **b** Studies including intensive care unit non-survivors only
  - Length of hospital stay: no RCTs reported on the mean length of hospital stay; however, six observational studies (1244 patients) [15, 16, 18, 19, 27, 32] did not find a difference in the length of hospital stay with the use of communication tools (MD −4.48 days, 95 % CI −9.11, 0.14, *p* = 0.06, very low-quality evidence), with marked heterogeneity (*I*^*2*^ = 78 %) (Fig. 9). One RCT (108 patients) reported the mean length of hospital stay in the subgroup of ICU non-survivors, finding no difference in the length of stay (median number of days, 23 vs. 21, *p* = 0.74) [14]

**Fig. 9:**
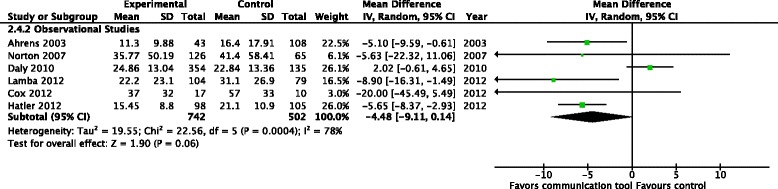
Health care resource utilization - mean length of hospital stay (days), Studies including both intensive care unit survivors and non-survivors
  - Financial costs: no RCTs, but five observational studies (670 patients) reported on the effects of the communication tools on financial costs [15, 17, 18, 20, 32], finding a reduction in hospital costs (SMD *−*0.30, 95 % CI *−*0.49, *−*0.11, p = 0.002, very low-quality evidence), with no evidence of statistical heterogeneity (*I*^2^ = 26 %) (Fig. 10). One RCT (108 patients) [14] reported financial costs amongst the subgroup of ICU non-survivors, finding no differences in health care costs (US$167,350 vs. US$164,670, *p* = 0.92). Two observational studies (124 patients) [17, 29] also found no differences in this subgroup (SMD −0.76, 95 % CI −1.57, 0.04, *p* = 0.06)

**Fig. 10:**
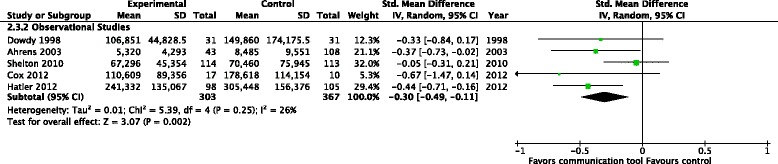
Health care resource utilization - financial costs, Studies including both intensive care unit survivors and non-survivors
5. Patient and family acceptability of the intervention: only two RCTs reported measures of the acceptability of the interventions, both finding the majority of SDMs would accept the use of the communication tool for EoL decision-making again in the future [23, 24]. One observational study of a video decision-support tool also reported that a majority of patients were comfortable with the intervention, found it helpful, and would recommended it to others [30]

## Discussion

Given the centrality of EoL decision-making to the care provided in the ICU, it is surprising that our review found so few studies evaluating the use of structured communication tools to assist SDMs and clinicians with this process. Only very low-quality evidence (from one RCT with a high risk of bias and four observational studies) was found that evaluated whether such tools increase the number of documented goals-of-care discussions, indicating that future studies are very likely to alter our estimates of effect. We found low-quality evidence that the use of structured communication tools does not increase the number of patients with documented code status or DNR status, or decisions to withdraw or withhold treatment. Equally surprising is that the use of communication tools, in comparison to usual care, had minimal to no effect in the studies we found that reported on these outcomes. This may be because the studies lacked sufficient power to find a true difference, the tools themselves were ineffective, the tools have variable efficacy without a significant *class effect* for their use, or because there is minimal room for measurable improvements beyond standard care once the patient is in the ICU. If the latter is true, it may be more effective to direct structured interventions earlier in the course of a patient’s care, before they are in the ICU, when there is a greater opportunity for such interventions to help patients and families create documented care plans, and improve the concordance between the care they receive, and the care they wish to receive. 

The one area where significant improvements with the use of structured communication tools was demonstrated was in health care resource utilization, although the quality of evidence was again very low. The existing evidence suggests that the use of such tools may decrease the number of days of mechanical ventilation, length of ICU stay, number of hospital days, and financial costs of care. These results were not seen in the exploratory subgroup analysis of ICU non-survivors, in which similar reductions in health care utilization were not demonstrated.

The mechanisms by which these interventions could decrease health care resource utilization without increasing the number of patients with documented code status or withholding or withdrawal of treatments is unclear. The most obvious possibility is that the studies that demonstrated a decrease in resource use were not the same studies that failed to find improvements in documented goals-of-care discussions, code status documentation, and decisions to withdraw or withhold therapies, and thus, heterogeneity between the studies may account for the differences. Or, it may be that in the studies evaluating these tools, while there actually were increases in the number of decisions to withhold or withdraw life-sustaining therapies, such decisions were poorly documented. If there is a clear consensus between SDMs and clinicians that the current ICU level of care is futile or unwanted, clinicians may fail to document resuscitation status and decisions to withdraw or withhold treatment, whereas in conflict-charged situations, documentation may be extensive. Another possible explanation for decreased resource use is that the use of communication tools may result in less aggressive, less expensive care, in ways that are not always considered to be withdrawing or withholding of life-sustaining therapies (e.g., fewer diagnostic tests and invasive procedures, or the use of one-way extubation, with rapid transfer out of ICU).

Overall, the low quality of evidence suggests that more high-quality randomized trials are needed to determine whether the use of structured communication tools to assist SDMs and clinicians with EoL decision-making has a major effect upon outcomes that are important to patients. Given the considerable experience ICU clinicians have in assisting SDMs with EoL decision-making, it may be reasonable to focus such studies on patient populations for which extra assistance would be needed. For instance, high-quality randomized trials of simple structured communication tools would be of value in ICU patients with conflict over goals of care, or in long-stay ICU patients. We would recommend that such studies report outcomes related to three domains: patient-level outcomes (e.g., documented code status, withdrawal or withholding of treatment, and mortality); family-level outcomes (satisfaction, and long-term mental health outcomes, including depression and anxiety), and system-level outcomes (resource use, including bed use), given that decisions about whether ICUs should implement structured communication tools to assist EoL decision-making should ideally take all three domains into account. Alternatively, it may be worth focusing more efforts on communication tools at earlier points in a patient’s care trajectory, such as in the ambulatory care setting, where the decisions made have the potential for greater impact on the care patients receive.

### Strengths

The strength of our study lies in its broad search strategy to find communication tools to assist in EoL decision-making. This allowed us to capture the full range of tools available to ICU clinicians, including traditional decision aids, structured meeting plans, educational interventions, the use of consulting services, and complex quality improvement programs. Further strengths of our study include: the rigorous search strategies; the input of two separate authors in assessing studies for screening, eligibility, and risk of bias, with secondary checking and verification of data extraction; and our use of GRADE to assess the overall quality of evidence for each outcome.

### Limitations

Our study has limitations related both to its methods, and to the studies we retrieved. First, our use of very broad inclusion criteria resulted in difficulty in identifying studies that evaluated interventions related to EoL decision-making. Extensive review of abstracts, study methods, and outcomes was often required to determine whether or not a study intervention was indeed a communication tool designed to assist in EoL decision-making, or a communication tool for another purpose (e.g*.*, not all palliative care consultations have the purpose of assisting decision-making; instead, many have the purpose of providing emotional comfort and symptom control). By not limiting our review to one specific type of intervention, it became more difficult to identify the studies we wanted to include; given this difficulty, it is possible that our review failed to identify some potentially relevant articles, despite our rigorous search.

Our second limitation was related to the outcomes reported in the studies we found. Although documented code status and decisions to withdraw and withhold treatments were commonly reported, they are ultimately surrogate measurements for unknown patient wishes. Ideally, studies would be able to directly assess patient wishes for care and assess concordance between patient preferences and care plans, and patient preferences for care and the care actually received. We recognize the fact that many patients may not have a completed advance directive or may not have discussed EoL preferences with their SDMs; however, we believe that reporting concordance between patient wishes and the actual care received at the EoL would still be an important, even if imperfect, outcome for future studies to report.

The third limitation of our review is the limited number of studies retrieved, which were varied in quality and intervention type. We found very few RCTs that reported on our outcomes of interest, with the result that one trial by Curtis et al., considered to be at substantial risk of bias, given the variability between clusters, lack of adjustment for cluster design for some outcomes, and loss of a cluster, was responsible for much of the randomized data in our primary outcomes. The quality of evidence for all of our outcomes is of very low to low quality according to GRADE, meaning our estimated effects are very likely to be affected by future studies.

## Conclusions

We found very low-quality evidence that the use of structured communication tools increases the number of documented goals-of-care discussions and low-quality evidence that they do not affect the number of patients with documented code status/DNR forms or decisions to withdraw/withhold life-sustaining treatments. We also found very low-quality evidence that the use of structured communication tools results in reduced health care resource utilization compared to usual care. More high-quality RCTs are required to evaluate the effects of structured communication tools to facilitate EoL decision-making in the ICU upon system-level, family-level, and patient-level outcomes, including concordance between patient wishes for care and the care received at the end of life.

## Key messages

- Many studies of structured communication tools to improve end-of-life decision-making in adult ICU patients have been published
- The evidence that such interventions increase the documentation of goals-of-care discussions and code status, or withdrawal and withholding of life-sustaining treatments is low to very low in quality
- Future studies of simple interventions in targeted ICU populations are needed, and should report upon patient-level, family-level, and system-level outcomes

## Additional file

Additional file 1: Appendix: Electronic search strategies. (DOC 72 kb)

## Abbreviations

CI: confidence interval
DNR: do not resuscitate
EoL: end of life
GRADE: Grading of Recommendations Assessment, Development, and Evaluation
HCP: health care provider
ICU: intensive care unit
MD: mean difference
RCT: randomized controlled trial
RR: relative risk
SDM: substitute decision-makers
SMD: standardized mean difference
